# Nematicidal Potential of *Purpureocillium takamizusanense* PMEPF27 Against Motile *Bursaphelenchus rainulfi* In Vitro

**DOI:** 10.3390/microorganisms14030714

**Published:** 2026-03-22

**Authors:** Yuh Tzean, Elena Gamboa Chen, Xiao-Yu Wei, I-En Shih, Hui-Yu Hsu, Ya-Zhen Xu, Ying-Hong Lin, Meng-Ling Wu, Tai-Yuan Chen, Yung-Yu Yang, Jen-Chih Chen

**Affiliations:** 1 Department of Plant Pathology and Microbiology, National Taiwan University, Taipei 106319, Taiwan; yuhtzean@ntu.edu.tw (Y.T.); d03642007@ntu.edu.tw (E.G.C.); huiyuxu18@gmail.com (H.-Y.H.); 2 Institute of Biotechnology, National Taiwan University, Taipei 106319, Taiwan; 3 Department of Plant Medicine, National Pingtung University of Science and Technology, Neipu 912301, Taiwan; sherrywei1214@gmail.com (X.-Y.W.); iannnnn712010@gmail.com (I.-E.S.); koji860206@gmail.com (Y.-Z.X.); pmyhlin@mail.npust.edu.tw (Y.-H.L.); 4 Division of Forest Protection, Taiwan Forestry Research Institute, Taipei 10066, Taiwan; mlw@tfri.gov.tw; 5 Kaohsiung District Agricultural Research and Extension Station, Ministry of Agriculture, Changzhi 908126, Taiwan; taiyuan@mail.kdais.gov.tw

**Keywords:** biological control, entomopathogenic fungi, fungus-nematode interaction, in vitro bioassay, pine-associated nematodes, scanning electron microscopy

## Abstract

*Bursaphelenchus rainulfi* is a pine-associated, non-pathogenic nematode that serves as a motile comparative species for evaluating nematophagous fungi. We investigated the in vitro biocontrol activity of *Purpureocillium takamizusanense* strain PMEPF27, originally isolated from insect cadavers in Taiwan, against mixed motile stages of *B. rainulfi*. Identity of the fungus was confirmed by morphology and *ITS*/*EF-1α* sequencing. Nematodes were exposed in liquid suspension to PMEPF27 conidia, with sterile water as the negative control and fluopyram as the positive control. Mortality was monitored over 7 days, and scanning electron microscopy was used to observe fungus–nematode interactions. PMEPF27 caused significantly higher mortality than the water control, reaching ~80% by day 7, and showed surface disruption of nematode cuticles, although no direct spore penetration was observed. These findings expand the nematophagous profile of *P. takamizusanense* beyond egg and sedentary stages, validate *B. rainulfi* as a motile comparative species in pine-nematode bioassays, and highlight PMEPF27 as a promising candidate for biocontrol development.

## 1. Introduction

Plant-parasitic nematodes (PPNs) are among the most destructive pests in agriculture and forestry, causing an estimated 12–13% reduction in global crop yields annually [1,2]. The pine wood nematode (PWN), *Bursaphelenchus xylophilus*, is particularly notorious as the causal agent of pine wilt disease (PWD) and is considered one of the most damaging forest pathogens worldwide [3]. Initially believed to be native to North America, the devastating impact of PWN was first documented in Japan during the early 20th century [4,5], and the nematode later made its way to Europe, first impacting Portugal and later spreading to Spain [6]. Because *B. xylophilus* is a quarantine organism in many countries, work with this pathogen is restricted and requires strict phytosanitary measures [7].

Current control measures for PWN and other PPNs rely heavily on chemical nematicides and phytosanitary interventions, like fumigation or burning of infested wood [8,9,10,11]. However, these approaches raise environmental and safety concerns, and their effectiveness is limited by cost, labor, and the potential for nematicide resistance [12]. There is an urgent need for sustainable, ecologically friendly nematode management strategies [13]. Consequently, there is a strong need for alternative, sustainable approaches and for laboratory models that allow mechanistic study of nematode–microbe interactions without the regulatory burden of working directly with quarantine nematodes [14].

Biological control has emerged as a promising pillar of integrated pest management (IPM), utilizing the natural antagonism between microorganisms and pests to achieve suppression [15]. Among the various microorganisms explored, nematophagous fungi have received considerable attention due to their diverse mechanisms of action, which include parasitism and the secretion of cuticle-degrading enzymes [16,17]. The genus *Purpureocillium*, formerly categorized under *Paecilomyces*, contains several species recognized for their robust nematophagous properties [18,19]. *Purpureocillium lilacinum* is perhaps the most well-known member, having been extensively commercialized for its ability to parasitize the eggs and sedentary stages of various nematodes [20,21]. The mechanisms of nematode inhibition by *P. lilacinum* include the production of cuticle-degrading enzymes such as proteases, chitinases, and lipases that facilitate hyphal penetration of nematode eggs and cuticles [22,23]. Additionally, *P. lilacinum* produces secondary metabolites with nematicidal and egg-hatching inhibitory effects [24,25].

In contrast, *P. takamizusanense* has been much less studied. First described as the anamorph of a *Cordyceps* teleomorph (*Isaria takamizusanensis*), it is recognized as an emerging entomopathogenic fungus in Asia [26,27]. Recent reports indicate that *P. takamizusanense* can infect diverse insect hosts and may exhibit plant growth-promoting traits, such as phosphate solubilization [26]. The genome of *P. takamizusanense* has been sequenced, revealing 11,855 protein-coding genes and 36 biosynthetic gene clusters that may contribute to bioinsecticidal activity [28]. Whether this species also possesses nematophagous capabilities remains largely unexplored.

Previous research on *Purpureocillium* biocontrol has largely concentrated on nematode eggs or sedentary stages, whereas comparatively less is known about activity toward motile vermiform stages. Evaluating pathogenicity against actively moving nematodes is essential for assessing the true biocontrol potential of these fungi. In the present study, *B. rainulfi* was selected as a model organism to examine the nematicidal capacity of *P. takamizusanense* strain PMEPF27. *B. rainulfi* is a pine-associated species originally described from decaying pine wood in Malaysia and subsequently reported in China and Taiwan [29,30]. Pathogenicity assays have demonstrated that it does not induce pine wilt disease [30], yet it co-occurs with other pine-associated nematodes and can serve as a reliable surrogate in laboratory bioassays. Furthermore, interactions of *B. rainulfi* with nematophagous fungi, including susceptibility to *Esteya vermicola* infection in vitro [31], reinforce its suitability as a model for fungus–nematode studies.

Here, we use *B. rainulfi* as a non-pathogenic motile model to investigate the nematophagous potential of *P. takamizusanense* PMEPF27 under controlled in vitro conditions. The objectives of this study were to (i) confirm the identity of strain PMEPF27 using morphological and molecular approaches, (ii) quantify its effect on motile *B. rainulfi* survival over time in comparison with a chemical nematicide control, and (iii) characterize fungus–nematode surface interactions by scanning electron microscopy (SEM) to generate hypotheses about possible killing mechanisms. To contextualize whether the observed activity reflects strain-specific or broader EPF-associated effects, we additionally included *Beauveria bassiana* PMEPF23 as a taxonomically distinct entomopathogenic fungal comparative control. By combining a non-pathogenic pine-associated nematode with an entomopathogenic fungus that shows nematicidal activity, we establish a tractable model system for dissecting fungal factors involved in nematode killing without the need to manipulate quarantine nematodes in the laboratory.

This study establishes a safe and tractable experimental model for investigating fungus–nematode interactions using a non-pathogenic, pine-associated motile nematode under controlled in vitro conditions. By demonstrating that *P. takamizusanense* can increase the mortality of motile nematodes, our findings expand the currently limited understanding of nematode-targeting activity in this species. These results contribute to a deeper understanding of fungal-nematode interactions and may inform the development of sustainable, biologically based strategies for nematode management.

## 2. Materials and Methods

### 2.1. Preparation of Bursaphelenchus rainulfi

*Bursaphelenchus rainulfi* was obtained from Dr. Pei-Chen Chen (Department of Plant Pathology, National Chung Hsing University, Taiwan). The nematodes were maintained on *Alternaria* sp. colonies cultured on potato dextrose agar (PDA), as described for this species [30]. *Alternaria* sp. colonies were cut using a circular puncher (diameter 1 cm) and subcultured on PDA, then incubated at 28 °C in the dark for 7 days. After 7 days, *B. rainulfi* were transferred onto *Alternaria* sp. colonies and cultured in the dark at 28 °C for 14 days. The *B. rainulfi* culture medium was washed with 3 mL of sterile water to prepare a nematode suspension. Each biological replicate in the experimental set contained approximately 50 nematodes.

### 2.2. Cultivation of Entomopathogenic Fungi (EPF)

The entomopathogenic fungal (EPF) strains used in the experiments were PMEPF27 (*Purpureocillium takamizusanense*) (PMEPF27) and PMEPF23 (*Beauveria bassiana*). PMEPF23 was isolated from a lepidopteran larva collected in Heping District, Taichung City, Taiwan. PMEPF27 was isolated from *Tessaratoma papillosa* collected in Laopi Village, Neipu Township, Pingtung County, Taiwan.

For conidial production, each EPF strain was cultured on PDA at 28 °C in the dark for 14 days. After incubation, the EPF culture medium was washed with 3 mL of sterile water, and a triangular glass rod was used to prepare spore suspensions. Spore concentrations were determined using a hemocytometer and adjusted to 1 × 10^6^ conidia mL^−1^ for bioassays.

### 2.3. Morphological Observation of PMEPF27

For microscopy observation of sporulation structures of PMEPF27, the fungal strain was cultured on water agar (WA) at 28 °C in the dark for 14 days. A circular piece (diameter 1 cm) of the mycelial medium was cut and placed on a slide, and the structures were observed using a microscope (Zeiss Axio Scope, Jena, Germany). Colony characteristics were documented on PDA after 7 days of incubation at 28 °C, including colony morphology, pigmentation, texture, and radial growth rate.

### 2.4. Molecular Identification of Fungal Isolate

PMEPF27 and PMEPF23 colonies were cultured on WA medium for 14 days. The mycelium was ground to a powder with liquid nitrogen, and 100 mg of the powdered sample was transferred to a 1.5 mL microcentrifuge tube. Fungal genomic DNA was isolated using the Plant DNA Mini Kit (LabPrep, Taipei, Taiwan) according to the manufacturer’s instructions. Molecular identification of fungi was conducted using *ITS* and *EF-1α* gene primers (Supplementary Table S1). The *ITS* region was amplified using primers ITS1 (5′-TCCGTAGGTGAACCTGCGG-3′) and ITS4 (5′-TCCTCCGCTTATTGATATGC-3′) [32]. The *EF-1α* gene was amplified using primers EF1-983F (5′-GCYCCYGGHCAYCGTGAYTTYAT-3′) and EF1-2218R (5′-ATGACACCRACRGCRACRGTYTG-3′) [33]. PCR was performed in 25 μL reactions containing 1× PCR buffer, 2.5 mM MgCl_2_, 0.2 mM dNTPs, 0.4 μM of each primer, 1 U Taq polymerase, and approximately 50 ng of template DNA. PCR cycling conditions were: initial denaturation at 95 °C for 5 min; 35 cycles of 95 °C for 30 s, 55 °C for 30 s, and 72 °C for 1 min; and final extension at 72 °C for 10 min. PCR products were purified using the GEL/PCR Purification mini Kit (FavorPrep™, Ping-Tung, Taiwan) and sequenced by Tri-I Biotech Inc., New Taipei City, Taiwan. Sequences were deposited in GenBank (PMEFP27 *ITS*: PZ092910; *EF-1α*: PZ111818).

### 2.5. Phylogenetic Analyses

Phylogenetic analyses of PMEPF27 were performed using the *ITS* and *EF-1α* sequences, along with reference strains, using Bayesian inference and maximum likelihood approaches. The accession numbers for the reference stains are listed in Supplementary Table S2. MrBayes inference analysis was conducted using MrBayes V3.2.7 [34,35,36]. Markov Chain Monte Carlo (MCMC) sampling was employed for 1,000,000 generations with lset nst = 6, while other parameters remained unchanged. Evolutionary analysis was performed using the Maximum Likelihood method and Tamura-Nei model [37]. The evolutionary history was inferred utilizing MEGA X [38].

### 2.6. In Vitro Nematode Mortality Assay of Entomopathogenic Fungi Against Bursaphelenchus rainulfi

A 6 cm plastic culture dish containing approximately 50 individual *B. rainulfi* in 3 mL sterile water was mixed with 3 mL of the corresponding conidial suspension (PMEPF27 or PMEPF23) adjusted to 1 × 10^6^ conidia mL^−1^, resulting in a final concentration of 5 × 10^5^ conidia mL^−1^. The culture dishes were tightly sealed with Parafilm to prevent evaporation and observed under a dissecting microscope at 24 h intervals for 7 days. The number of motile (living) and immotile (dead) nematodes was recorded. Nematodes were considered dead when they showed no movement after gentle agitation or mechanical stimulation with a fine probe. The 7-day observation period was selected to capture delayed mortality effects and to characterize the full time course of fungal activity, which can develop over multiple days.

In the negative control group, the spore suspension of either PMEPF27 or PMEPF23 was replaced with sterile water. In the positive control group, fluopyram (Velum^®^ Prime, Bayer CropScience, St. Louis, MO, USA), a succinate dehydrogenase inhibitor nematicide, was used. A 1500-fold dilution of fluopyram was mixed with an equal volume of nematode suspension, resulting in the manufacturer’s recommended concentration for nematode control (approximately 0.5 mg L^−1^ active ingredient). Each treatment group consisted of three biological replicates (*n* = 3). The experiment was repeated twice to confirm reproducibility (experimental sets 1 and 2). For each time point, mortality data were analyzed by one-way ANOVA followed by Tukey’s multiple-comparison test to evaluate differences among treatments (negative control, fluopyram, and fungal treatment). Statistical significance was defined at *p* < 0.05.

### 2.7. Scanning Electron Microscopy

The fungal-infected *B. rainulfi* were first prepared as described previously for the in vitro assay. Nematodes were collected by centrifugation (300 rpm, 3 min, 25 °C) and deposited at the bottom, which were then transferred onto the glass coverslips using 200 µL and 1000 µL pipettes. The samples were fixed in glutaraldehyde and stored at 4 °C for 1–2 h. Sample preparation for SEM, including dehydration and critical point drying, was conducted similarly according to the standard biological specimen protocols described previously [39]. Following incubation, samples were washed twice with PBS buffer for 15 min each. A graded series of alcohol concentrations (30%, 50%, 70%, 80%, 90%) was used for dehydration, 10 min each step, followed by two 15 min treatments with 100% alcohol, and then two 15 min treatments with acetone. Samples were then dried using a critical point dryer (CPD) (73 atm, 1 h 10 min, 37 °C). After drying, the coverslips were attached to metal stubs coated with carbon tape using forceps and sputter-coated using an ion sputter coater (7 atm, 180 s, 30–40 °C). Finally, the surface morphology of the nematodes and the fungal parasitism were examined using a scanning electron microscope (Hitachi, Tokyo, Japan).

## 3. Results

### 3.1. Growth Characteristics and Morphology

**Figure 1:**
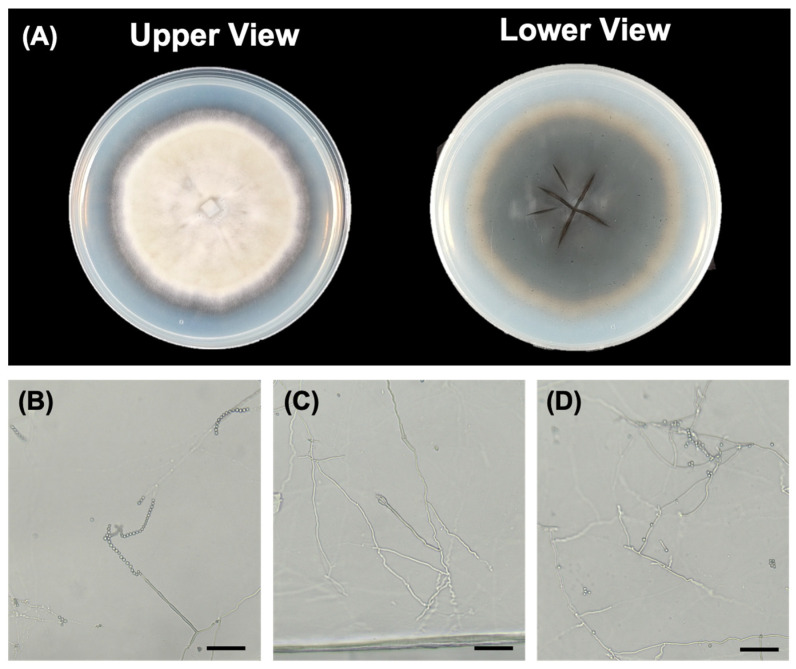
Colony morphology and conidiation of PMEPF27. (**A**) Colony appearance of PMEPF27 grown on potato dextrose agar (PDA), shown from the upper and lower (reverse) sides. (**B**–**D**) Light micrographs of asexual reproductive structures, showing phialides with conidia in chains (**B**), hyphae bearing conidiophores (**C**), and clustered conidiogenous structures with conidial chains (**D**). Scale bars = 10 μm (**B**–**D**).

Strain PMEPF27 grew readily on PDA, exhibiting a dense, velvety texture with a radially folded surface and white to purplish pigmentation at the colony edge (Figure 1A). The radial growth reached 1.81 ± 0.28 cm after 7 days of incubation at 28 °C. The reverse side of colonies showed cream to pale purple coloration.

Microscopic examination of PMEPF27 showed smooth-walled, single-celled conidia that were elliptical to cylindrical in shape, and arranged in long chains (Figure 1B–D). Conidia measured 2.5–4.0 μm in length and 1.5–2.5 μm in width. Hyphae were septate and branched. Conidiophores were erect and often formed clusters with a series of phialides (Figure 1B–D). These morphological characteristics were consistent with the description of *Purpureocillium* species [40,41].

### 3.2. Molecular Characterization of PMEPF27

To confirm the taxonomic identity of PMEPF27, the *internal transcribed spacer* (*ITS*) region and *elongation factor-1α* (*EF-1α*) gene regions were amplified and sequenced. The *ITS* sequence was 532 bp (GenBank: PZ092910) and the *EF-1α* sequence was 936 bp (GenBank: PZ11818). BLAST 2.17.0 analysis showed high sequence similarity of PMEFP27 to *P. takamizusanense* reference strains for both *ITS* and *EF-1α* (Supplementary Table S2).

**Figure 2:**
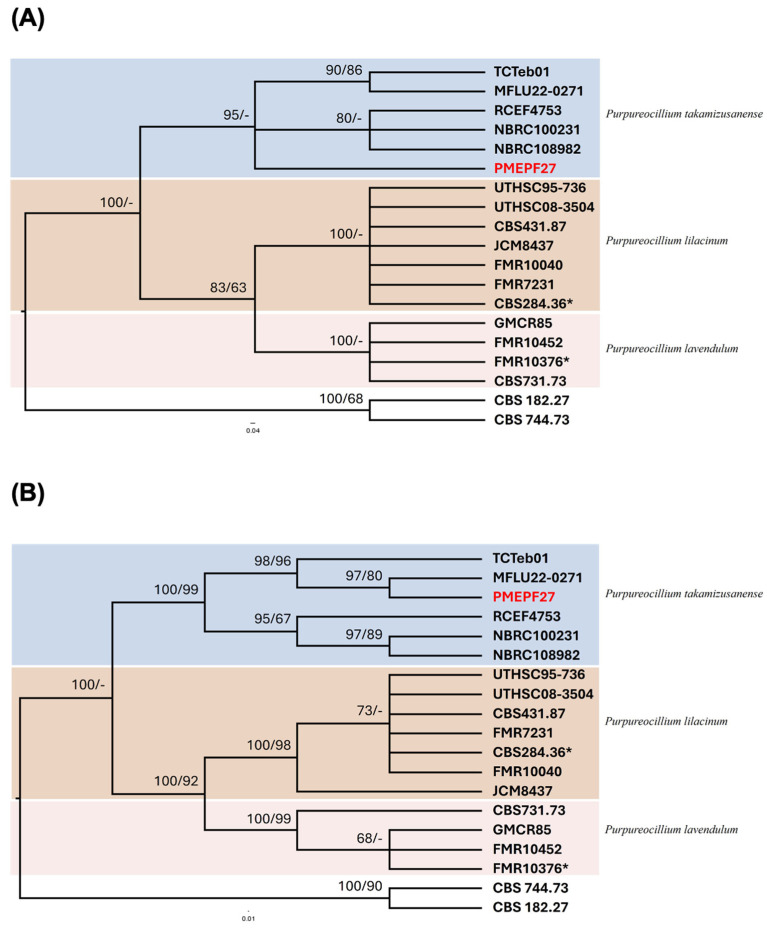
Phylogenetic tree depicting the relationship between PMEPF27 and related genera, constructed using the MrBayes analysis method based on *ITS* (**A**) and *EF-1α* (**B**) sequence data. Bayesian posterior probabilities and maximum-likelihood (ML) values, denoted as MrBayes/ML, are provided on each branch. The tree was inferred through Markov Chain Monte Carlo sampling for 1,000,000 iterations, with *Paecilomyces marquandii* and *Nomuraea atypicola* designated as outgroups. The asterisk (*) indicates the ex-type strain. The strain identified in this study, PMEPF27, is highlighted in red. The bar represents genetic distance.

Phylogenetic analysis using Bayesian inference (MrBayes) and maximum likelihood (ML) methods showed three main distinct clades comprising *P. takamizusanense*, *P. lilacinum*, and *P. lavendulum* (Figure 2). PMEPF27 clustered within the clade comprising *P. takamizusanense* with high statistical support in both trees. In the *ITS*-based tree, PMEPF27 grouped with *P. takamizusanense* reference strains with 100% Bayesian posterior probability and 98% ML bootstrap support (Figure 2A). Similarly, in the *EF-1α*-based tree, PMEPF27 formed a well-supported clade with *P. takamizusanense* strains, showing 100% Bayesian posterior probability and 97% ML bootstrap support (Figure 2B). These results identified PMEPF27 as *P. takamizusanense*.

### 3.3. Pathogenicity of PMEPF27 Against Bursaphelenchus rainulfi

**Figure 3:**
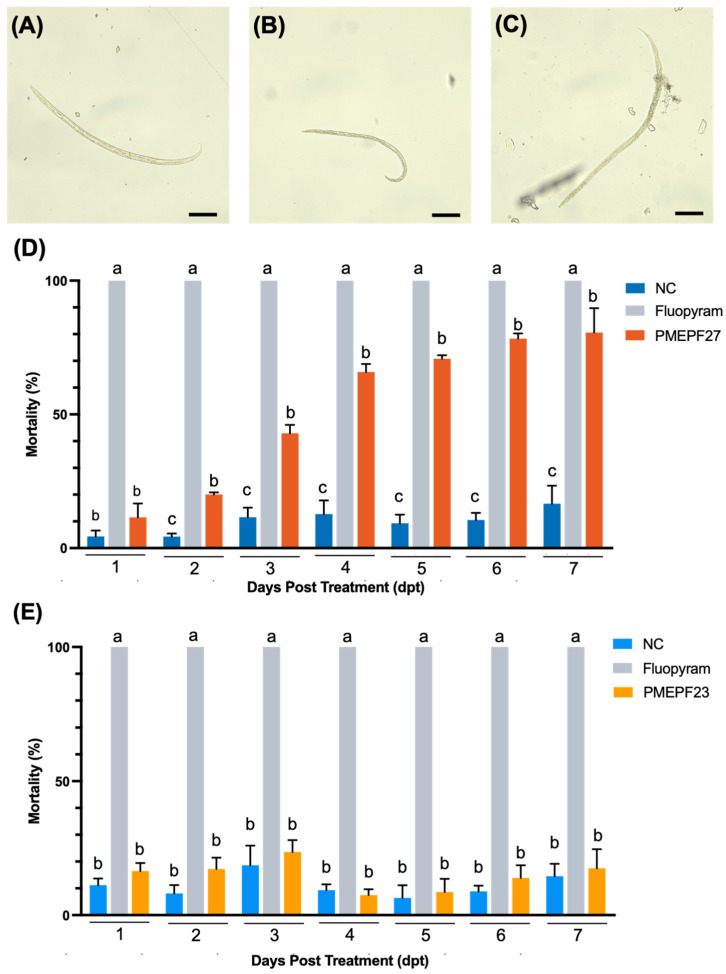
Effects of entomopathogenic fungi on *Bursaphelenchus rainulfi* mortality in vitro. (**A**–**C**) Representative micrographs of *B. rainulfi* after exposure to sterile water (negative control; **A**), fluopyram (chemical control; **B**), or PMEPF27 (*Purpureocillium takamizusanense*; **C**). Scale bars = 100 μm. (**D**) Mortality (%) of *B. rainulfi* after treatment with sterile water, fluopyram, or PMEPF27 over 1–7 days post-treatment (dpt). (**E**) Mortality (%) of *B. rainulfi* after treatment with sterile water, fluopyram, or PMEPF23 (*Beauveria bassiana*) over 1–7 dpt. Bars represent mean ± SD (*n* = 3). For each dpt, treatment means were compared by one-way ANOVA followed by Tukey’s multiple-comparison test (*p* < 0.05). Different letters indicate significant differences among treatments within the same dpt.

**Figure 4:**
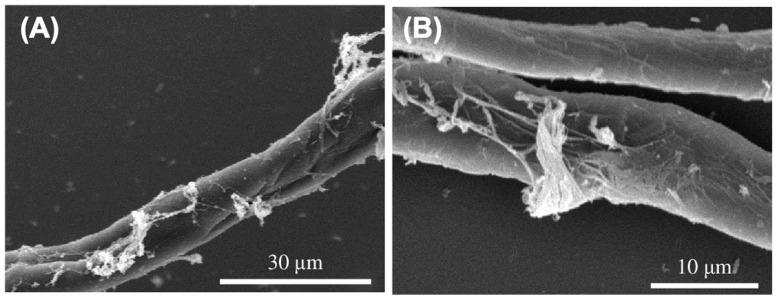
Scanning electron microscopy of *Bursaphelenchus rainulfi* following exposure to PMEPF27. (**A**) Shows a nematode cuticle with visible signs of degradation and adherence of extracellular structures at a magnification of scale bar = 30 µm. (**B**) A close-up view of a breached cuticle segment with extensive structural disintegration and fungal hyphae attachment. Scale bar = 10 µm.

Mortality of motile *B. rainulfi* was quantified following exposure to conidial suspensions of PMEPF27 (*P. takamizusanense*) (Figure 3A–D). To contextualize whether the observed mortality patterns were specific to PMEPF27 or could also be observed with another EPF taxon under the same assay format, PMEPF23 (*B. bassiana*) was evaluated in a parallel assay as a taxonomically distinct entomopathogenic fungal control (Figure 4). In each assay, sterile water served as the negative control (NC) and fluopyram served as the chemical positive control. Fluopyram resulted in 100% mortality by 1 day post-treatment (dpt), the first observation time point, whereas mortality in NC remained low throughout the observation period (Figure 3D and Figure 4).

PMEPF27 exposure produced a clear time-dependent increase in mortality relative to NC (Figure 3D). Mortality increased progressively over the observation period and reached high levels at later time points (5–7 dpt). The independent repeat (Experimental Set 2) showed a similar temporal pattern (Supplementary Figure S1A), supporting reproducibility. One-way ANOVA followed by Tukey’s multiple-comparison test indicated significantly higher mortality in the PMEPF27 treatment than in NC at multiple time points (*p* < 0.05; Figure 3D).

In the PMEPF23 assay, mortality remained comparatively low over time (Figure 4). Although immotile nematodes were observed in the PMEPF23 treatment, the overall mortality profile did not show a sustained increase relative to NC under the multiple-comparison analysis. The independent repeat (Experimental Set 2) is provided in Supplementary Figure S1B. Together, these results indicate that EPF strains differed in the magnitude and temporal dynamics of mortality against motile *B. rainulfi* under the assay conditions, with PMEPF27 showing the stronger time-dependent effect.

### 3.4. Pathogenicity of PMEPF27 Against Bursaphelenchus rainulfi as Revealed by Scanning Electron Microscopy (SEM)

To further analyze the interaction of PMEPF27 and *B. rainulfi*, scanning electron microscopy (SEM) was used to observed *B. rainulfi* that was exposed to PMEPF27. Based on the observation under SEM, by 72 h post-exposure, marked degradation of the nematode cuticle was observed (Figure 4). Although several nematodes displayed eroded, disrupted, and breached cuticles, no PMEPF27 spores were detected adhering to the nematodes at the examined time point.

## 4. Discussion

This study demonstrates the strong in vitro biocontrol activity of *P. takamizusanense* PMEPF27 against motile *B. rainulfi*, a pine-associated nematode that serves as a surrogate species for laboratory evaluation of fungal effects relevant to pinewood nematode systems. Previous research on this fungus has focused predominantly on its entomopathogenic properties and potential plant growth-promoting capabilities [26,27,42]. In Taiwan, *P. takamizusanense* was first documented as an entomopathogenic fungus infecting *Tessaratoma papillosa* adults [41], and the species has been reported to exhibit activity against agricultural pests from multiple insect orders [26]. Our findings expand the currently recognized functional scope of *P. takamizusanense* and motivate further examination of the interactions with nematodes.

*P. lilacinum* has been extensively studied for nematode control over past decades [22,23,43,44,45,46]. A number of studies emphasize egg parasitism where fungi penetrate egg shells or cuticles and then consume the nematode contents [47]. The fungus produces an array of hydrolytic enzymes, including serine proteases, subtilisins, chitinases, and lipases, that degrade the chitin-rich egg shells and protein-rich cuticles of nematodes [22,23,48,49]. Whole-genome analysis has confirmed that *P. lilacinum* possesses large sets of these hydrolytic enzymes, with serine proteases specifically up-regulated in the presence of nematode eggs [19,28]. For example, overexpression of a serine protease gene in *P. lilacinum* significantly enhanced virulence against *Meloidogyne incognita* eggs [50]. In the case of motile nematodes, *P. lilacinum* can also infect second-stage juveniles (J2) by enzymatic digestion of the cuticle [51,52], though many studies have concentrated on egg infection since eggs are stationary targets.

In our assay, PMEPF27 caused a time-dependent increase in mortality of motile nematodes over 5–7 days, whereas fluopyram produced complete mortality by the first observation time point. Although PMEPF27 acted more slowly than the chemical control, it produced substantial mortality by later time points. The time course observed here is consistent with report for other *Purpureocillium* spp. affecting motile juveniles over multi-day intervals, including *P. lavendulum* against *M. incognita* J2 [53], and *P. lilacinum*, which typically kills *Meloidogyne* eggs and females within 5–7 days [19,49,54]. While chemicals like fluopyram offer rapid nematicidal effect, biological agents like *P. lilacinum* are increasingly recommended for sequential application with chemical nematicides to provide sustained control and improved yields [54].

In addition, culture-derived compounds from *P. lavendulum* have been reported to exhibit nematicidal or egg-hatching inhibitory effects [53,55]. Recent chemo-profiling has identified specific metabolites responsible for this activity, including 5-methoxymethyl-1H-pyrrole-2-carboxaldehyde from *P. lavendulum* [53], as well as hydroxylated fatty acids and steroids [55]. In *P. lilacinum*, organic acids, phenolic compounds, and fatty acids such as myristic and lauric acid have been correlated with high J2 mortality [24,25]. Together, these findings support the view that *Purpureocillium* spp. may affect nematodes through a combination of processes, although the relative contributions are likely strain- and context dependent.

The SEM observations documented cuticle damage in PMEPF27-treated nematodes; however, we did not conidia directly attached to nematode surfaces or appressoria-like penetration structures at the examined time points. These observations are compatible with, but do not demonstrate, a role extracellular enzymes and metabolites. Secondary metabolite diversity and secreted enzymes, including leucinostatins, have been documented in *Purpureocillium* spp. and are linked to nematode antagonisms in other systems [18,22,23,24,25,56]. Genomic resources available for *P. takamizusanense* also suggest the potential for substantial repertoire of secreted proteins and secondary-metabolite biosynthetic gene clusters [28]. Accordingly, an important next step will be to couple time-resolved microscopy with assays of extracellular enzyme activities and culture-filtrate bioactivity, followed by chemical profiling and gene-level interrogation, to identify which factors are responsible for PMEPF27-induced mortality.

Our study also supports *B. rainulfi* as a potential comparative model organism for evaluating fungal effects on pine-associated nematodes. *B. rainulfi* is phylogenetically related to the pathogenic *B. xylophilus* but does not cause pine wilt disease [30], making it a safer alternative for laboratory studies that do not require quarantine facilities. In Taiwan, pathogenicity assays on *Pinus thunbergii* indicate that *B. rainulfi* did not induce wilting symptoms under tested conditions [30]. The species has been reported from pine wood in Malaysia, China, and Taiwan and co-occurs with other *Bursaphelenchus* spp. in pine ecosystems [29,30,57]. Previous studies also showed that *B. rainulfi* is susceptible to infection by the nematophagous fungus *Esteya vermicola* [31], demonstrating its utility for fungus–nematode interaction studies. Future studies could directly compare PMEFP27 effects on *B. rainulfi* and *B. xylophilus* to PMEPF27 to determine whether the biocontrol efficacy extends to the pathogenic species.

Our comparison of *P. takamizusanense* PMEPF27 and *B. bassiana* PMEPF23 revealed marked differences in nematicidal activity. Including PMEPF23 as a taxonomically distinct entomopathogenic fungal control provided context for EPF-associated effects under the same assay format. Under our conditions, PMEPF23 produced comparatively low mortality and did not show a sustained increase relative to the negative control, whereas PMEPF27 produced a stronger time-dependent effect. Although *Beauveria* species have been reported to exhibit nematicidal activity against *B. xylophilus* in vitro and *B. mucronatus* in vivo [58,59], nematode-antagonistic effects appear to be strain- and context-dependent rather than universal within the genus. In contrast, the stronger activity observed for PMEPF27 is consistent with the biology of *Purpureocillium* species, which commonly inhabit soil environments where nematode interactions are frequent [18,22]. Furthermore, the ability of *Purpureocillium* isolates to tolerate water stress and varying temperature ranges supports their capacity to function effectively under variable agroecological conditions [12,18]. Together, these findings highlight the importance of standardized assays and empirical screening when selecting fungal candidates for nematode biocontrol.

This study provides evidence that *P. takamizusanense* PMEPF27 can affect motile pine-associated nematodes in vitro. These findings expand the currently recognized functional breadth of *B. rainulfi* and establish a tractable platform for future mechanistic studies. Further work should prioritize identification of causal factors underlying mortality, evaluation against *B. xylophilus* under appropriate containment, and assessment of formulation and delivery strategies relevant to pine systems.

## 5. Conclusions

*P. takamizusanense* PMEPF27 increased the mortality of motile *B. rainulfi* under controlled in vitro conditions and was associated with surface cuticle damage observable by SEM. Molecular and phylogenetic analyses identified PMEPF27 as *P. takamizusanense*. The use of *B. rainulfi* as a non-quarantine, pine-associated motile model provides a tractable platform for screening and hypothesis generation relevant to pinewood nematode systems. Future work should determine the causal factors underlying PMEPF27-associated mortality through targeted assays of culture filtrates, extracellular enzyme activities, and secreted metabolites, and should evaluate efficacy against *B. xylophilus* under appropriate containment.

## Data Availability

The original contributions presented in this study are included in the article/Supplementary Materials. Further inquiries can be directed to the corresponding authors.

## Supplementary Materials

The following supporting information can be downloaded at: https://www.mdpi.com/article/10.3390/microorganisms14030714/s1, Table S1: Primers used for molecular identification of PMEPF27 strain; Table S2: Species used for phylogenetic analysis of PMEPF27; Figure S1: Independent repeat (Experimental Set 2) of *Bursaphelenchus rainulfi* mortality assays. References [32,33] are cited in Supplementary Materials.

## References

[1] Singh S., Singh B., Singh A. (2015). Nematodes: A threat to sustainability of agriculture. Proc. Environ. Sci..

[2] Decraemer W., Hunt D.J. (2006). Structure and classification. Plant Nematology.

[3] Pires D., Vicente C.S., Inácio M.L., Mota M. (2022). The Potential of *Esteya* spp. for the Biocontrol of the Pinewood Nematode, *Bursaphelenchus xylophilus*. Microorganisms.

[4] Linit M. (1988). Nemtaode-vector relationships in the pine wilt disease system. J. Nematol..

[5] Kikuchi T., Cotton J.A., Dalzell J.J., Hasegawa K., Kanzaki N., McVeigh P., Takanashi T., Tsai I.J., Assefa S.A., Cock P.J. (2011). Genomic insights into the origin of parasitism in the emerging plant pathogen *Bursaphelenchus xylophilus*. PLoS Pathog..

[6] Mota M.M., Braasch H., Bravo M.A., Penas A.C., Burgermeister W., Metge K., Sousa E. (1999). First report of *Bursaphelenchus xylophilus* in Portugal and in Europe. Nematology.

[7] Dwinell L.D. (1997). The pinewood nematode: Regulation and mitigation. Ann. Rev. Phytopathol..

[8] Takai K., Suzuki T., Kawazu K. (2003). Development and preventative effect against pine wilt disease of a novel liquid formulation of emamectin benzoate. Pest Manag. Sci..

[9] Kwon T.-S., Song M.-Y., Shin S.-C., Park Y.-S. (2005). Effects of aerial insecticide sprays on ant communities to control pine wilt disease in Korean pine forests. App. Entomol. Zool..

[10] Zhao B.G. (2008). Pine wilt disease in China. Pine Wilt Disease.

[11] Bonifácio L.F., Sousa E., Naves P., Inacio M.L., Henriques J., Mota M., Barbosa P., Drinkall M.J., Buckley S. (2014). Efficacy of sulfuryl fluoride against the pinewood nematode, *Bursaphelenchus xylophilus* (Nematoda: Aphelenchidae), in *Pinus pinaster* boards. Pest Manag. Sci..

[12] Rigobelo E.C., Nicodemo D., Babalola O.O., Desoignies N. (2024). *Purpureocillium lilacinum* as an agent of nematode control and plant growth-promoting fungi. Agronomy.

[13] Poveda J., Abril-Urias P., Escobar C. (2020). Biological control of plant-parasitic nematodes by filamentous fungi inducers of resistance: *Trichoderma*, mycorrhizal and endophytic fungi. Front. Microbiol..

[14] Zhang Y., Li S., Li H., Wang R., Zhang K.-Q., Xu J. (2020). Fungi–nematode interactions: Diversity, ecology, and biocontrol prospects in agriculture. J. Fungi.

[15] Topalović O., Hussain M., Heuer H. (2020). Plants and associated soil microbiota cooperatively suppress plant-parasitic nematodes. Front. Microbiol..

[16] Dou G., Yan D.-H. (2022). Research progress on biocontrol of pine wilt disease by microorganisms. Forests.

[17] Ayaz M., Zhao J.-T., Zhao W., Chi Y.-K., Ali Q., Ali F., Khan A.R., Yu Q., Yu J.-W., Wu W.-C. (2024). Biocontrol of plant parasitic nematodes by bacteria and fungi: A multi-omics approach for the exploration of novel nematicides in sustainable agriculture. Front. Microbiol..

[18] Girardi N.S., Sosa A.L., Etcheverry M.G., Passone M.A. (2022). In vitro characterization bioassays of the nematophagous fungus *Purpureocillium lilacinum*: Evaluation on growth, extracellular enzymes, mycotoxins and survival in the surrounding agroecosystem of tomato. Fungal Biol..

[19] Prasad P., Varshney D., Adholeya A. (2015). Whole genome annotation and comparative genomic analyses of bio-control fungus *Purpureocillium lilacinum*. BMC Genom..

[20] James B., Godonou I., Atcha C., Baimey H., Adango E., Boulga J., Goudegnon E. (2006). Healthy vegetables through participatory IPM in peri-urban areas of Benin. Summary of Activities and Achievements.

[21] Kerry B.R. (2000). Rhizosphere interactions and the exploitation of microbial agents for the biological control of plant-parasitic nematodes. Ann. Rev. Phytopathol..

[22] Moreno-Gavíra A., Huertas V., Diánez F., Sánchez-Montesinos B., Santos M. (2020). *Paecilomyces* and its importance in the biological control of agricultural pests and diseases. Plants.

[23] Bali G.K., Singh S.K., Maurya D.K., Wani F.J., Pandit R.S. (2022). Morphological and molecular identification of the entomopathogenic fungus *Purpureocillium lilacinum* and its virulence against *Tuta absoluta* (Meyrick) (Lepidoptera: Gelechiidae) larvae and pupae. Egypt. J. Biol. Pest Control.

[24] Patidar P., Prasad L., Sagar S., Sirohi A., Saharan M.S., Dhillon M.K., Singh V.K., Bag T.K. (2024). Chemo-profiling of *Purpureocillium lilacinum* and *Paecilomyces variotii* isolates using GC-MS analysis, and evaluation of their metabolites against *M. incognita*. PLoS ONE.

[25] Sharma A., Gupta A., Dalela M., Sharma S., Sayyed R., Enshasy H.A.E., Elsayed E.A. (2020). Linking organic metabolites as produced by *Purpureocillium lilacinum* 6029 cultured on Karanja deoiled cake medium for the sustainable management of root-knot nematodes. Sustainability.

[26] Lo P.-H., El-Sayid Abdrabo K.A., Nai Y.-S., Lu H.-l., Huang Y.-T. (2025). Versatile entomopathogenic activity of *Purpureocillium takamizusanense* against diverse agricultural pests. bioRxiv.

[27] Zhang Z., Chen W., Liang J., Zhang L., Han Y., Huang J., Liang Z. (2022). Revealing the non-overlapping characteristics between original centers and genetic diversity of *Purpureocillium lilacinum*. Fungal Ecol..

[28] Nguyen N.-H., Tamura T., Shimizu K. (2022). Draft genome sequence of *Purpureocillium takamizusanense*, a potential bioinsecticide. Microbiol. Resour. Announc..

[29] Braasch H., Burgermeister W. (2002). *Bursaphelenchus rainulfi* sp. n. (Nematoda: Parasitaphelenchidae), first record of the genus Bursaphelenchus Fuchs, 1937 from Malaysia. Nematology.

[30] Chang C., Chen P. (2020). Identification of *Bursaphelenchus rainulfi* (Nematoda: Parasitaphelenchidae), a new record pine wood nematode species in Taiwan and its pathogenicity. J. Plant Med..

[31] Wang X., Wang T., Wang J., Guan T., Li H. (2014). Morphological, molecular and biological characterization of *Esteya vermicola*, a nematophagous fungus isolated from intercepted wood packing materials exported from Brazil. Mycoscience.

[32] White T.J., Bruns T., Lee S., Taylor J. (1990). Amplification and direct sequencing of fungal ribosomal RNA genes for phylogenetics. PCR Protoc. A Guide Methods Appl..

[33] Rehner S.A., Buckley E. (2005). A Beauveria phylogeny inferred from nuclear *ITS* and *EF1-α* sequences: Evidence for cryptic diversification and links to Cordyceps teleomorphs. Mycologia.

[34] Huelsenbeck J.P., Ronquist F. (2001). MRBAYES: Bayesian inference of phylogenetic trees. Bioinformatics.

[35] Ronquist F., Huelsenbeck J.P. (2003). MrBayes 3: Bayesian phylogenetic inference under mixed models. Bioinformatics.

[36] Ronquist F., Teslenko M., Van Der Mark P., Ayres D.L., Darling A., Höhna S., Larget B., Liu L., Suchard M.A., Huelsenbeck J.P. (2012). MrBayes 3.2: Efficient Bayesian phylogenetic inference and model choice across a large model space. System. Biol..

[37] Tamura K., Nei M. (1993). Estimation of the number of nucleotide substitutions in the control region of mitochondrial DNA in humans and chimpanzees. Mol. Biol. Evol..

[38] Kumar S., Stecher G., Li M., Knyaz C., Tamura K. (2018). MEGA X: Molecular evolutionary genetics analysis across computing platforms. Mol. Biol. Evol..

[39] Bozzola J.J., Russell L.D. (1999). Electron Microscopy: Principles and Techniques for Biologists.

[40] Luangsa-Ard J., Houbraken J., van Doorn T., Hong S.-B., Borman A.M., Hywel-Jones N.L., Samson R.A. (2011). Purpureocillium, a new genus for the medically important *Paecilomyces lilacinus*. FEMS Microbiol. Lett..

[41] Lo P., Yu Y., Pai K. (2019). First report of *Purpureocillium takamizusanense* as an entomopathogenic fungus infecting *Tessaratoma papillosa* (Drury) in Taiwan. J. Plant Med..

[42] Lopez-Nuñez R., Prieto-Rubio J., Bautista I., Lidón-Cerezuela A.L., Valverde-Urrea M., Lopez-Moya F., Lopez-Llorca L.V. (2025). Chitosan reduces naturally occurring plant pathogenic fungi and increases nematophagous fungus *Purpureocillium* in soil under field conditions. Front. Agron..

[43] Lan X., Zhang J., Zong Z., Ma Q., Wang Y. (2017). Evaluation of the biocontrol potential of *Purpureocillium lilacinum* QLP12 against Verticillium dahliae in eggplant. BioMed Res. Int..

[44] Elsherbiny E.A., Taher M.A., Elsebai M.F. (2019). Activity of *Purpureocillium lilacinum* filtrates on biochemical characteristics of Sclerotinia sclerotiorum and induction of defense responses in common bean. Eur. J. Plant Pathol..

[45] Li Y., Zhang C., Zhong M., Hu S., Cui Y., Fang J., Yu X. (2024). Revealing the metabolic potential and environmental adaptation of nematophagous fungus, *Purpureocillium lilacinum*, derived from hadal sediment. Front. Microbiol..

[46] de Freitas Soares F.E., Sufiate B.L., de Queiroz J.H. (2018). Nematophagous fungi: Far beyond the endoparasite, predator and ovicidal groups. Agric. Nat. Resour..

[47] Khan A., Williams K.L., Nevalainen H.K. (2006). Infection of plant-parasitic nematodes by *Paecilomyces lilacinus* and *Monacrosporium lysipagum*. BioControl.

[48] Giné A., Sorribas F.J. (2017). Effect of plant resistance and BioAct WG (*Purpureocillium lilacinum* strain 251) on *Meloidogyne incognita* in a tomato–cucumber rotation in a greenhouse. Pest Manag. Sci..

[49] El-Marzoky A.M., Ali M.A., Elnahal A.S., Abuljadayel D.A., Alkherb W.A., Moustafa M., Alshaharni M.O., Abd El-Aal E.M. (2025). The Combination Effect of *Purpureocillium lilacinum* Strain (AUMC 10620) and Avermectin (B1a and B1b) on Control Citrus Nematode *Tylenchulus* semipenetrans (Cobb) Under Laboratory and Field Conditions. Biology.

[50] Wang J., Wang J., Liu F., Pan C. (2010). Enhancing the virulence of *Paecilomyces lilacinus* against *Meloidogyne incognita* eggs by overexpression of a serine protease. Biotechnol. Lett..

[51] Kiewnick S., Sikora R. (2006). Biological control of the root-knot nematode Meloidogyne incognita by Paecilomyces lilacinus strain 251. Biol. Control.

[52] Khan M., Tanaka K. (2023). *Purpureocillium lilacinum* for plant growth promotion and biocontrol against root-knot nematodes infecting eggplant. PLoS ONE.

[53] Bao Z.-X., Liu R., Li C.-Q., Pan X.-R., Zhao P.-J. (2022). Pathogenicity and Metabolites of *Purpureocillium lavendulum* YMF1. 00683 against *Meloidogyne incognita*. Pathogens.

[54] Khan A., Khan A., Ali A., Fatima S., Siddiqui M.A. (2023). Root-knot nematodes (*Meloidogyne* spp.): Biology, plant-nematode interactions and their environmentally benign management strategies. Gesunde Pflanz..

[55] Liu R., Bao Z.-X., Li G.-H., Li C.-Q., Wang S.-L., Pan X.-R., Zhang K.-Q., Zhao P.-J. (2022). Identification of nematicidal metabolites from *Purpureocillium lavendulum*. Microorganisms.

[56] Chen W., Hu Q. (2021). Secondary metabolites of *Purpureocillium lilacinum*. Molecules.

[57] Jiang L.-q., Li X.-q., Zheng J.-w. (2007). First record of *Bursaphelenchus rainulfi* on pine trees from eastern China and its phylogenetic relationship with intro-genus species. J. Zhejiang Univ. Sci. B.

[58] Sánchez-Gómez T., Harte S.J., Zamora P., Bareyre M., Díez J.J., Herrero B., Niño-Sánchez J., Martín-García J. (2023). Nematicidal effect of Beauveria species and the mycotoxin beauvericin against pinewood nematode *Bursaphelenchus xylophilus*. Front. For. Glob. Change.

[59] Sánchez-Gómez T., Zamora P., Díez J.J., Herrero B., Poveda J., Martín-García J. (2025). Using *Bursaphelenchus mucronatus* to demonstrate the potential nematicidal effect of *Beauveria bassiana* on pine wood nematode (*Bursaphelenchus xylophilus*) under in vivo conditions. Ann. For. Sci..

