# Supplementary material for: Nematicidal Potential of Purpureocillium takamizusanense PMEPF27 Against Motile Bursaphelenchus rainulfi In Vitro
Source: Microorganisms. 2026 Mar 22;14(3):714. doi: 10.3390/microorganisms14030714 (PMC13028671; doi:10.3390/microorganisms14030714)
Supplement: Supplementary file 1 [file microorganisms-14-00714-s001.zip › microorganisms-4169542-supplementary.pdf]

Supplementary Tables and Figures

Supplementary Table S1. Primers used for molecular identification of PMEPF27 strain.

Primer Name	Sequence	Amplified Region	Reference
**ITS1**	**TCCGTAGGTGAACCTGCGG**	**rRNA**	**White et al., 1990**
**ITS4**	**TCCTCCGCTTATTGATATGC**		
**EF1-983F**	**GCYCCYGGHCAYCGTGAYTTYAT**	**EF-1α**	**Rehner and Buckley, 2005**
**EF1-2218R**	**ATGACACCRACRGCRACRGTYTG**		

Supplementary Table S2. Type strain species use for phylogenetic analysis of PMEPF27.

Species	Strain	ITS GenBank no.
Purpureocillium takamizusanense	MFLU22-0271	OQ127363
Purpureocillium takamizusanense	NBRC108982	LC008204
Purpureocillium takamizusanense	NBRC100231	LC008203
Purpureocillium takamizusanense	NBRC110232	LC008205
Purpureocillium takamizusanense	RCEF4753	MT568629
Purpureocillium takamizusanense	TCTeb01	MK592777
Purpureocillium lilacinum	CBS284.36	FR734101
Purpureocillium lilacinum	CBS431.87	AY634188
Purpureocillium lilacinum	FMR7231	FR734085
Purpureocillium lilacinum	FMR10040	FR734092
Purpureocillium lilacinum	JCM8437	FR734103
Purpureocillium lilacinum	UTHSC08-3504	FR734098
Purpureocillium lilacinum	UTHSC95-736	FR734100
Purpureocillium lavendulum	CBS731.73	HE792981
Purpureocillium lavendulum	FMR10376	FR734106
Purpureocillium lavendulum	FMR10452	FR734107
Purpureocillium lavendulum	GMCR85	LT220721
Paecilomyces marquand	CBS 182.27	AY624193
Nomuraea atypicola	CBS 744.73	GU980041

(A)

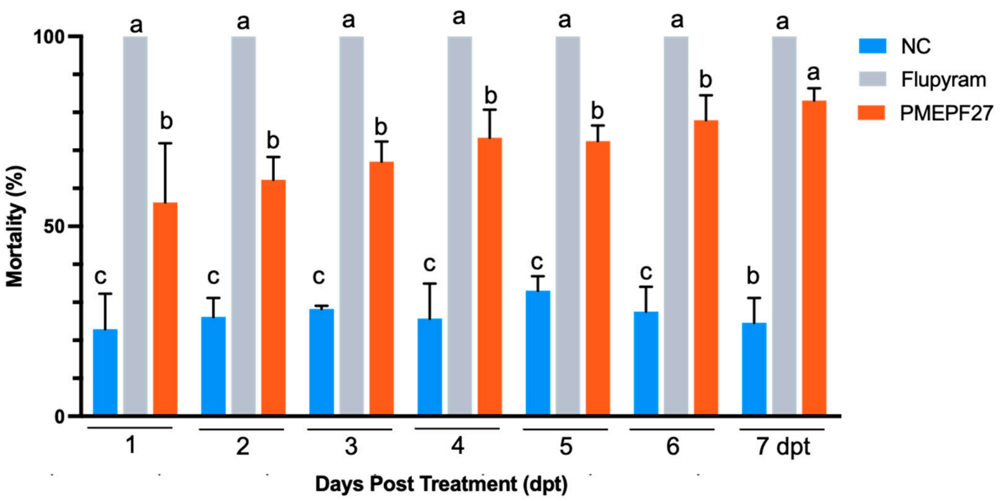

(B)

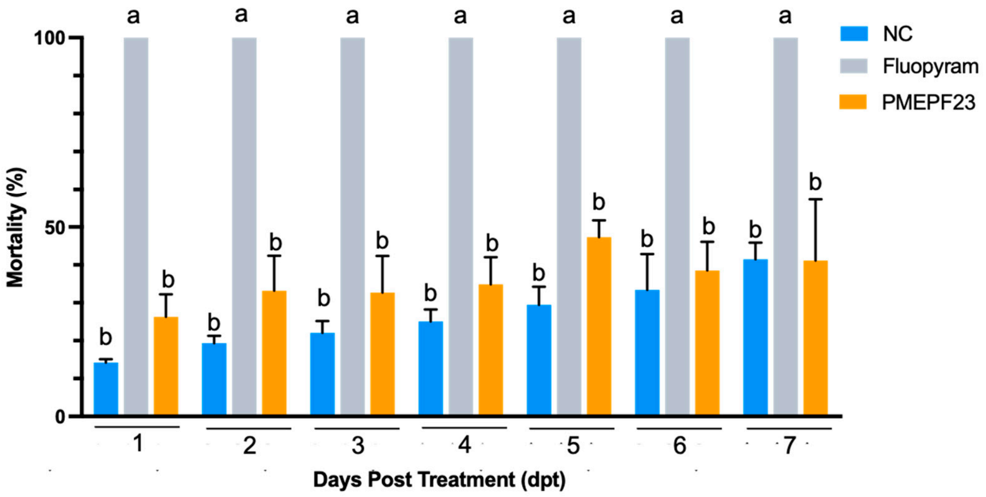

Supplementary Figure S1. Independent repeat (Experimental Set 2) of *Bursaphelenchus rainulfi* mortality assays. (**A**) Mortality (%) of motile *B. rainulfi* following exposure to sterile water (negative control), fluopyram (positive chemical control), or PMEPF27 (*Purpureocillium takamizusanense*) over 1–7 days post-treatment (dpt). (**B**) Mortality (%) of motile *B. rainulfi* following exposure to sterile water, fluopyram, or PMEPF23 (*Beauveria bassiana*) over 1–7 dpt. Bars represent mean ± SD ($n = 3$). Within each time point, treatment means were compared by one-way ANOVA followed by Tukey's multiple-comparison test ($p < 0.05$). Different letters indicate significant differences among treatments within the same dpt.
